# Evaluating the impact of childhood BMI on the risk of coronavirus disease 2019: A Mendelian randomization study

**DOI:** 10.1515/med-2024-0923

**Published:** 2024-03-15

**Authors:** Yuan Liu, Yujian Miu, Ningjie Zhang, Wenhao Yu, Yu Chen, Jianli Zhang, Bo Zhang

**Affiliations:** Department of Infectious Disease Control, Wenling Center for Disease Control and Prevention, Taizhou 317500, China; Department of Orthopedics, First People’s Hospital of Wenling Affiliated to Wenzhou Medical University, 190 Taiping South Road, Taizhou 317500, China

**Keywords:** childhood, body mass index, Mendelian randomization, COVID-19

## Abstract

**Introduction:**

Although the correlation between childhood obesity and coronavirus disease 2019 (COVID-19) has been explored, the causality of these remains uncertain. Thus, we conducted a two-sample Mendelian randomization (MR) analysis to identify the causal association.

**Methods:**

Instrumental variables of childhood obesity were selected from genome-wide association study involving 61,111 Europeans. Besides, we collected summary statistics of different COVID-19 outcomes (susceptibility, hospitalization, and severity) from genome-wide association study including more than 2 million Europeans. The inverse-variance weighted was applied to assess the causality of childhood obesity with COVID-19. Furthermore, we replicated the above association based on another study.

**Results:**

Inverse-variance weighted results suggested that childhood obesity promoted the COVID-19 susceptibility but has not been validated in other approaches. For hospitalization and severity of COVID-19, we found that childhood obesity, respectively, increased 30 and 38% risk (*P* < 0.001), which were consistent in other MR approaches.

**Discussion:**

Our study provides evidence for a causal relationship between childhood BMI and COVID-19 which is consistent with previous studies. Though these explanations are biologically plausible, further studies are warranted to elucidate the role of these.

**Conclusions:**

Our study suggests the potential causal associations of childhood obesity with COVID-19, especially hospitalization and severity of COVID-19.

## 1 Introduction

The coronavirus disease 2019 (COVID-19) pandemic, caused by the highly infectious SARS-CoV-2, has rapidly emerged as a significant global health crisis [1]. The clinical presentation of COVID-19 varies in severity, ranging from asymptomatic infection to critical illness characterized by respiratory failure, septic shock, and/or multiple organ dysfunction [2]. As on March 7, 2023, the pandemic has resulted in 760 million COVID-19 cases and more than 6.87 million deaths worldwide [3]. Hence, it is crucial to identify modifiable risk factors associated with COVID-19 to enhance public health policies and clinical decision-making.

As for childhood obesity, a pressing public health concern, remains a prominent issue as the prevalence of overweight or obese children in the United States surpasses one-third, with this trend persistently on the rise [4]. Though the virus affects people of all ages, previous studies have shown that individuals with overweight or obese are at a higher risk of developing severe symptoms and worse prognosis of COVID-19 [5–7]. Some studies have shown that childhood obesity is associated with a higher risk and severe symptoms of COVID-19 in children [8,9]. For example, a cross-sectional study including 46 pediatric intensive care units (ICU) has shown that prehospital comorbidities such as obesity seem to be an important factor for symptom in children [10]. However, no significant correlation was observed between obesity and the need for ICU admission [11].

**Figure 1:**
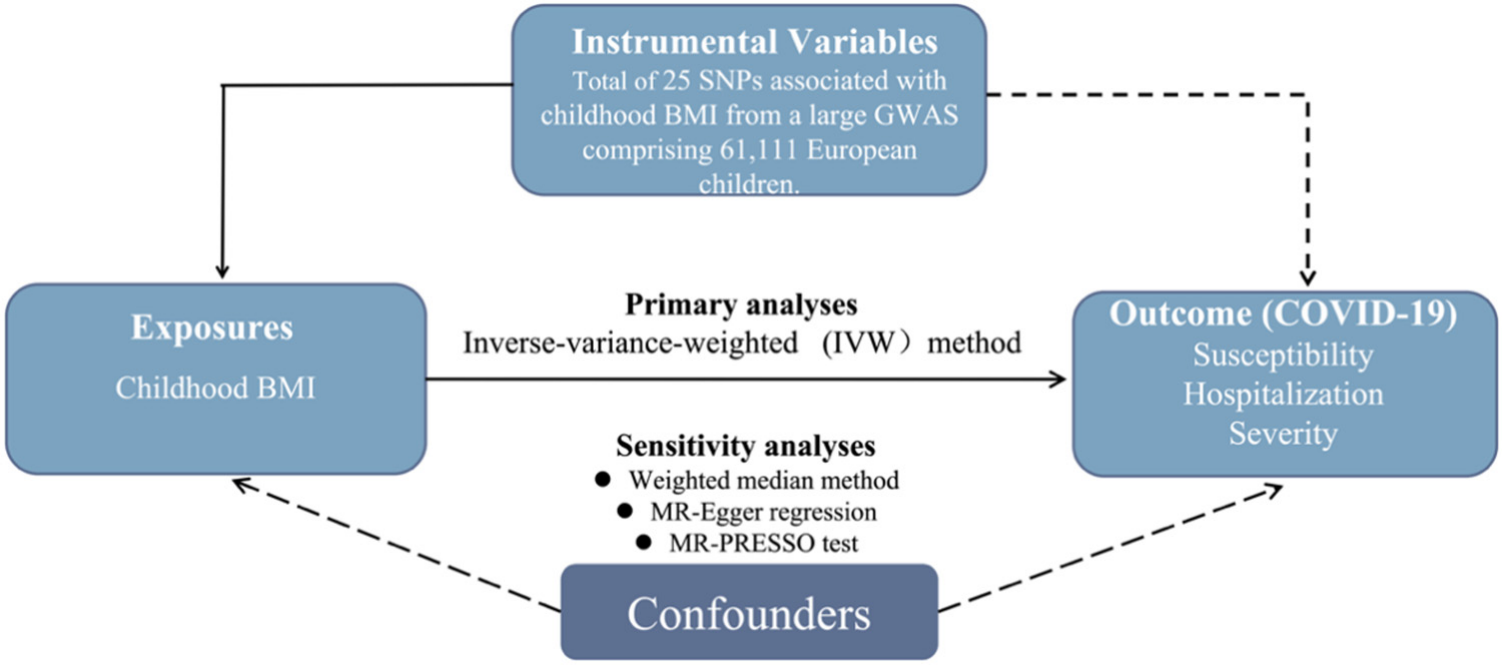
Schematic presentation in this MR analysis. BMI, body mass index; COVID-19, the coronavirus disease 2019; GWAS, genome-wide association study; MR, Mendelian randomization; MR-PRESSO, MR pleiotropy residual sum and outlier test; SNP, single nucleotide polymorphism.

Since conventional observational studies have several limitations, such as confounding and reverse causality, which might weaken the reliability of the results to a certain extent. Mendelian randomization (MR) is an approach to explore the potential causal relations between environmental exposures and diseases by instrumental variable (IV) which utilizes genetic variants [12,13]. Since genotypes are presumed to be randomly allocated in the process of gamete formation, the introduction of IV model to a large extent solves the problem of confounding in observational studies [14,15]. Moreover, since genotypes precede disease onset, reverse causation can be avoided in MR studies. Therefore, in this present study, we performed a two-sample MR study using the largest summary data to investigate the potential association between childhood body mass index (BMI) and risk of COVID-19. The overall study design is shown in Figure 1.

## 2 Methods

### 2.1 Outcome data source

The COVID-19 Host Genetics Initiative is a global collaborative effort in genetics research, aimed at identifying the genetic underpinnings of COVID-19 susceptibility and severity outcomes. In order to achieve this objective, researchers from diverse regions of the world have amalgamated individual-level clinical and genetic data and have conducted genome-wide association studies (GWAS). Summary data were procured from version seven (https://www.covid19hg.org, assessed on December 22, 2022) of the COVID-19 Host Genetics Initiative, and exclusively comprised participants of European descent. The original investigators of the COVID-19 GWAS data have undertaken adjustments for age, gender, principal components, and study-specific covariates. This study categorized COVID-19 outcomes into three distinct groups, namely susceptibility (1,22,616 COVID-19 cases vs 2,475,240 controls), hospitalization (32,519 COVID-19 patients requiring hospitalization vs 2,062,805 controls), and severity (13,769 confirmed COVID-19 cases with very severe respiratory manifestations vs 1,072,442 controls).

### 2.2 Selection of IV

MR studies rely on three key assumptions [15]. The first assumption is that the IVs are associated with the risk factor of interest. To ensure this, we used single nucleotide polymorphisms (SNPs) from independent loci associated with childhood BMI achieving genome-wide significance level (*P* < 5 × 10^−8^, *r* ^2^ < 0.1), which identified from the largest GWAS meta-analysis comprising 61,111 European children aged 2–10 years [16]. The second assumption is that the IVs are not associated with potential confounders of the exposure–outcome association. The third assumption is that the IVs influence the outcome only through the risk factor. To ensure this, we excluded potential pleiotropic SNPs and retained those solely associated with childhood BMI in the subsequent MR analyses. Furthermore, in order to validate the causal relationship between childhood BMI and COVID-19, we performed additional MR analysis using IVs obtained from another GWAS dataset, including 47,541 children [17]. This GWAS comprised 47,541 children and identified 15 loci associated with childhood BMI (*P* < 5 × 10^−8^).

### 2.3 Statistical analysis

First, the *F*-statistics was implemented to gauge the potency of IVs, as per the following formula: *F* = *R* ^2^ × (*N* − *k* − 1)/[*k* × (1 − *R* ^2^)], where *N* denotes the sample size, *k* refers to the number of chosen IVs, and *R* ^2^ represents the overall proportion of variance accounted for by the IVs, calculated based on the minor allele frequency and effect value of each IV [18,19]. An *F*-statistic value greater than 10 indicates a minimal likelihood of weak instrument bias; otherwise, an *F*-statistic value below 10 suggests that weak instrument bias may potentially impact the association between childhood BMI and COVID-19 [18,19].

The causal relationship between childhood BMI and COVID-19 was assessed by inverse variance weighting (IVW) method. The IVW method conducts a meta-analysis of Wald values, which involves dividing the beta coefficient of the SNP for the outcome by the beta coefficient of the SNP for the exposure [20]. To assess the heterogeneity among each, we used Cochran’s *Q* test which influenced the selection of appropriate model for the IVW analysis. If significant heterogeneity was detected (*P* < 0.05), random model was applied in IVW method; otherwise, fixed model was chosen [21]. In addition, the results obtained from the IVW method were validated through the utilization of additional methods, including the maximum likelihood, weighted median, and sample median method [18,22,23]. These complementary methods were employed to ensure the robustness and reliability of the causal relationship between the childhood BMI and the COVID-19.

We then used the MR-Egger regression method to evaluate potential directional pleiotropy by examining the intercept term [24]. Furthermore, to assess the level of horizontal pleiotropy among the IVs and outlier SNPs, MR multiplicity residuals and outliers (MR-PRESSO) was utilized [25]. Furthermore, the “leave-one-out” analysis was conducted by removing the IVs one by one, which used to assess the robustness of association.

Finally, the above MR-analysis was replicated utilizing other series IVs to verify the casual inference between childhood BMI and COVID-19. All analyses were based on the R version 4.5 and *P* < 0.05 was considered as statistically significant.


**Ethical approval:** The summary-level data of our MR study was approved by their Institutional Review Board (IRB) or an equivalent committee and written informed consent was obtained from all participants.

## 3 Results

A total of 23 SNPs were selected as IVs for subsequent MR analysis. The *F*-statistics of two series of IVs were 66.22 and 57.54, indicating that the selected variances were not affected by the weak instruments bias. More information of IVs in this study is presented in Tables S1 and S2. The Cochran’s *Q* test found that there was significant heterogeneity of IVs in the association between childhood BMI and COVID-19 susceptibility (*P* = 0.012) and hospitalization (*P* = 0.001) but did not find in the COVID-19 severity (*P* = 0.117). Thus, the random model of IVW was applied to estimate the casual association of childhood BMI with COVID-19 susceptibility and hospitalization and fixed model was used in the association between childhood BMI and COVID-19 severity (Table 1).

**Table 1:** MR analysis for the association between childhood BMI and COVID-19

Outcomes	Methods	Number of SNPs	OR	95% CI	*P*	*P* for intercept of MR-Egger	*P* for Cochran’s Q test	*P* for MR-PRESSO global test
COVID-19 susceptibility								
	IVW (random model)	23	1.07	1.02, 1.12	0.011		0.013	
	MR-Egger	23	1.06	0.90, 1.25	0.513	0.889		
	Weighted median	23	1.05	1.00, 1.11	0.071			
	Simple median	23	1.05	1.00, 1.12	0.071			
	Maximum-likelihood method	23	1.07	1.02, 1.13	0.010			
	MR-PRESSO (1 outliers)	22	1.05	1.02, 1.09	0.009			0.113
COVID-19 hospitalization								
	IVW (random model)	23	1.30	1.15, 1.46	<0.001		0.001	
	MR-Egger	23	1.46	1.00, 2.13	0.050	0.517		
	Weighted median	23	1.33	1.19, 1.50	<0.001			
	Simple median	23	1.33	1.18, 1.50	<0.001			
	Maximum-likelihood method	23	1.30	1.16, 1.47	<0.001			
	MR-PRESSO (1 outliers)	22	1.26	1.14, 1.38	<0.001			0.316
COVID-19 severity								
	IVW (fixed model)	23	1.38	1.22, 1.55	<0.001		0.118	
	MR-Egger	23	1.78	1.15, 2.76	0.010	0.224		
	Weighted median	23	1.47	1.23, 1.76	<0.001			
	Simple median	23	1.47	1.22, 1.77	<0.001			
	Maximum-likelihood method	23	1.39	1.20, 1.60	<0.001			
	MR-PRESSO (0 outliers)	23	1.38	1.20, 1.59	<0.001			0.139

**Figure 2:**
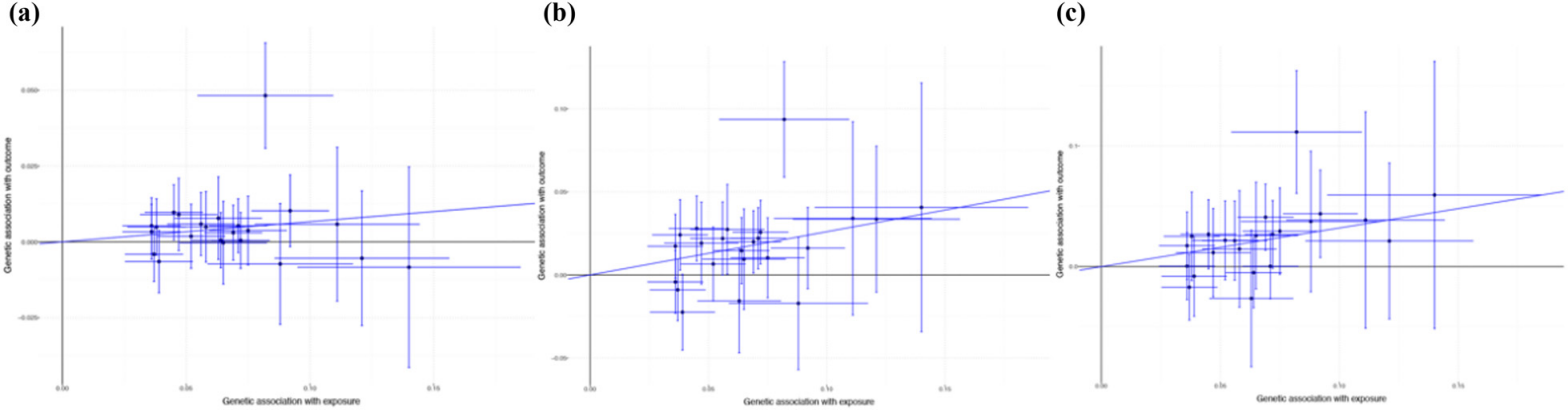
Casual association between childhood BMI and COVID-19 in the IVW method: (a) COVID-19 susceptibility, (b) COVID-19 hospitalization, and (c) COVID-19 severity. COVID-19, the coronavirus disease 2019.

IVW suggested that childhood BMI was positively associated with the COVID-19 susceptibility (odds ratio [OR]: 1.07, 95% confidence interval [CI], 1.02–1.12; *P =* 0.011, Figure 2a). Besides, there were similar effects of childhood BMI on COVID-19 hospitalization and severity in the IVW methods (Figure 2b and c). The maximum-likelihood method suggested that genetically predicted childhood BMI would increase COVID-19 risk (susceptibility: OR, 1.07, 95% CI, 1.02–1.13, *P* < 0.001; hospitalization: OR, 1.30, 95% CI, 1.16–1.47, *P* < 0.001; severity: OR, 1.39, 95% CI, 1.20–1.60, *P* < 0.001). Similar causal associations of childhood BMI with COVID-19 were observed in the sample median method and weighted median method (Table 1).

**Figure 3:**
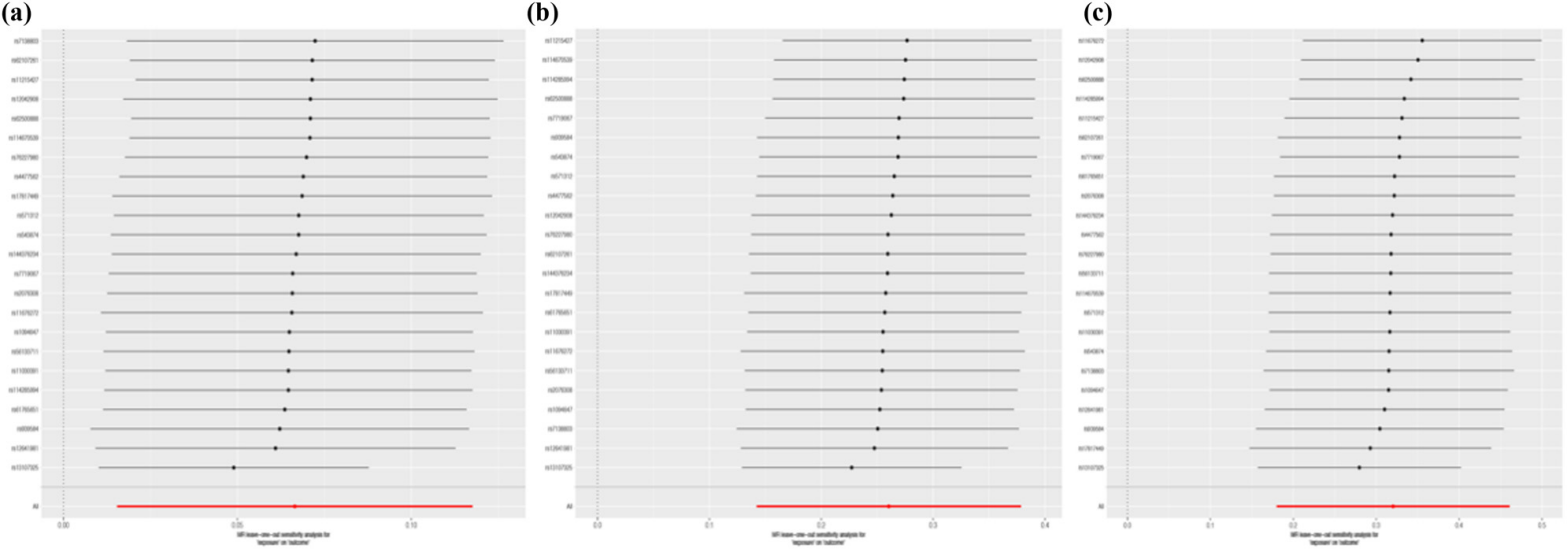
Casual association between childhood BMI and COVID-19 in the leave-one-out method: (a) COVID-19 susceptibility, (b) COVID-19 hospitalization, and (c) COVID-19 severity. COVID-19, the coronavirus disease 2019.

In the MR-Egger analysis, there were no effects of horizontal pleiotropy on the association between childhood BMI and COVID-19 (Table 1). MR-PRESSO detected one outlier SNPs in the association between childhood BMI and COVID-19 susceptibility and hospitalization, while casual association was consistent with IVW (Table 1). Besides, the leave-one-out suggested that the casual associations of childhood BMI with COVID-19 were not affected by single SNPs (Figure 3). For other series IVs, there was similar positive association between childhood BMI and COVID-19 (Table S3).

## 4 Discussion

This study provides evidence for a causal relationship between childhood BMI and COVID-19 susceptibility, hospitalization, and severity. These findings are consistent with previous studies that showed that individuals who were overweight or obese were at a higher risk of developing severe COVID-19 outcomes [8,9]. Evidence derived from a study utilizing national data on pediatric cases in Mexico revealed that children classified as obese exhibited a 39% higher likelihood of contracting SARS-CoV-2 infection [26]. Another retrospective cohort study also provided similar results that patients with obesity had longer adjusted length of stay (exponentiated parameter estimate 1.3; 95% CI: 1.1–1.5) compared to patients without obesity [27]. To clarify the causal relationship, we utilized GWAS data and MR analysis to confirm the conclusion that childhood BMI can increase the risk of COVID-19.

Consistently, our results supported a positive effect of childhood BMI on the development of COVID-19 and the results were consistent in the validation analysis. Besides, several studies have suggested potential biological mechanism involved in the pathogenesis of COVID-19. One potential mechanism by which childhood obesity may increase the risk of COVID-19 is through its impact on the immune system. Obesity has been shown to cause chronic low-grade inflammation by secreting several pro‐inflammatory cytokines, which can impair the immune system’s ability to fight infections that increase the risk for COVID‐19 [28]. In addition, obesity could result in altered lung mechanics and physiology, including altered topographical distribution of ventilation and reduced lung volumes especially in children [29,30]. Moreover, it is often associated with other comorbidities, such as diabetes, hypertension, and cardiovascular disease, which may also increase the risk of severe COVID-19 outcomes [31,32]. Though these explanations are biologically plausible, further studies are warranted to elucidate the role of childhood BMI on the development of COVID-19.

However, there are still some potential limitations to this study. First, it is unclear whether our findings can be extrapolated to other study populations, because data of our MR analysis are mainly based on participants of European ancestry. Second, limited to the summary-level data in our MR analyses, we cannot rule out the possibility of a nonlinear causal relationship of childhood BMI with the risk of COVID-19. Third, the features of childhood BMI, such as height and abdominal circumference, were not acquired which may be helpful in further classifying childhood BMI and broaden the findings. Despite these, our study still provided some crucial guidance for the prevention of COVID-19 in the population that had childhood obesity.

## 5 Conclusion

To the best of our knowledge, this study is the first MR analysis to investigate the causal association between childhood BMI and COVID-19 risk and provides important insights into it. The findings suggest that childhood BMI is a causal risk factor for COVID-19 hospitalization and severity. Thus, it is important to implement effective preventive measures to address childhood obesity, as it holds crucial implications for mitigating the impact of COVID-19 and other infectious diseases.

## Supplementary Material

Supplementary Table
