# Supplementary material for: Evaluating the impact of childhood BMI on the risk of coronavirus disease 2019: A Mendelian randomization study
Source: Open Med (Wars). 2024 Mar 15;19(1):20240923. doi: 10.1515/med-2024-0923 (PMC10996998; doi:10.1515/med-2024-0923)
Supplement: Supplementary Table [file med-2024-0923-sm.pdf]

Supplementary material

Table S1: The IVs information in the primary MR analysis

SNP	Childhood BMI				COVID-19 susceptibility					COVID-19 hospitalization					COVID-19 severity				
	EA	NEA	Beta	SE	EA	NEA	Beta	Se	*P*	EA	NEA	Beta	Se	*P*	EA	NEA	Beta	Se	*P*
rs1094647	G	A	0.038	0.006	A	G	-4.91×10^{-3}	4.73×10^{-3}	3.00×10^{-1}	A	G	-2.41×10^{-2}	1.08×10^{-2}	2.58×10^{-2}	A	G	-2.51×10^{-2}	1.87×10^{-2}	1.79×10^{-1}
rs11030391	A	G	0.036	0.006	G	A	-5.30×10^{-3}	4.70×10^{-3}	2.60×10^{-1}	G	A	-1.73×10^{-2}	9.80×10^{-3}	7.73×10^{-2}	G	A	-1.74×10^{-2}	1.43×10^{-2}	2.23×10^{-1}
rs11215427	G	C	0.039	0.007	C	G	6.50×10^{-3}	5.25×10^{-3}	2.16×10^{-1}	C	G	2.24×10^{-2}	1.16×10^{-2}	5.36×10^{-2}	C	G	7.92×10^{-3}	1.69×10^{-2}	6.40×10^{-1}
rs114285994	G	A	0.063	0.009	A	G	-7.79×10^{-3}	6.95×10^{-3}	2.62×10^{-1}	A	G	1.56×10^{-2}	1.60×10^{-2}	3.28×10^{-1}	A	G	2.63×10^{-2}	2.89×10^{-2}	3.62×10^{-1}
rs114670539	T	C	0.088	0.015	T	C	-7.29×10^{-3}	1.02×10^{-2}	4.74×10^{-1}	T	C	-1.71×10^{-2}	2.03×10^{-2}	4.01×10^{-1}	T	C	3.74×10^{-2}	2.97×10^{-2}	2.08×10^{-1}
rs116664060	C	G	0.049	0.009	NA	NA	NA	NA	NA	NA	NA	NA	NA	NA	NA	NA	NA	NA	NA
rs11676272	G	A	0.071	0.006	G	A	5.30×10^{-3}	4.54×10^{-3}	2.42×10^{-1}	G	A	2.21×10^{-2}	9.41×10^{-3}	1.89×10^{-2}	G	A	3.37×10^{-4}	1.38×10^{-2}	9.81×10^{-1}
rs12042908	A	G	0.064	0.006	G	A	-5.42×10^{-4}	4.65×10^{-3}	9.07×10^{-1}	G	A	-1.46×10^{-2}	1.02×10^{-2}	1.50×10^{-1}	G	A	4.98×10^{-3}	1.48×10^{-2}	7.37×10^{-1}
rs12641981	T	C	0.045	0.006	T	C	9.73×10^{-3}	4.64×10^{-3}	3.59×10^{-2}	T	C	2.80×10^{-2}	1.00×10^{-2}	5.17×10^{-3}	T	C	2.69×10^{-2}	1.46×10^{-2}	6.58×10^{-2}
rs13107325	T	C	0.082	0.014	T	C	4.82×10^{-2}	8.86×10^{-3}	5.14×10^{-8}	T	C	9.36×10^{-2}	1.77×10^{-2}	1.33×10^{-7}	T	C	1.12×10^{-1}	2.59×10^{-2}	1.71×10^{-5}
rs144376234	T	C	0.111	0.017	T	C	5.82×10^{-3}	1.30×10^{-2}	6.53×10^{-1}	T	C	3.40×10^{-2}	2.97×10^{-2}	2.51×10^{-1}	T	C	3.86×10^{-2}	4.57×10^{-2}	3.99×10^{-1}
rs17817449	G	T	0.069	0.006	G	T	3.02×10^{-3}	4.63×10^{-3}	5.13×10^{-1}	G	T	1.99×10^{-2}	9.57×10^{-3}	3.75×10^{-2}	G	T	4.10×10^{-2}	1.40×10^{-2}	3.43×10^{-3}
rs184566112	A	T	0.057	0.011	NA	NA	NA	NA	NA	NA	NA	NA	NA	NA	NA	NA	NA	NA	NA
rs2076308	C	G	0.058	0.008	C	G	4.95×10^{-3}	5.93×10^{-3}	4.04×10^{-1}	C	G	2.74×10^{-2}	1.38×10^{-2}	4.65×10^{-2}	C	G	1.47×10^{-2}	2.46×10^{-2}	5.51×10^{-1}
rs4477562	T	C	0.065	0.009	T	C	-2.95×10^{-4}	6.96×10^{-3}	9.66×10^{-1}	T	C	9.47×10^{-3}	1.54×10^{-2}	5.39×10^{-1}	T	C	2.57×10^{-2}	2.25×10^{-2}	2.54×10^{-1}
rs543874	G	A	0.075	0.008	G	A	3.75×10^{-3}	5.79×10^{-3}	5.17×10^{-1}	G	A	1.03×10^{-2}	1.23×10^{-2}	4.02×10^{-1}	G	A	2.94×10^{-2}	1.82×10^{-2}	1.07×10^{-1}
rs56133711	A	G	0.056	0.007	A	G	5.86×10^{-3}	5.31×10^{-3}	2.70×10^{-1}	A	G	2.20×10^{-2}	1.12×10^{-2}	5.05×10^{-2}	A	G	2.15×10^{-2}	1.68×10^{-2}	1.99×10^{-1}
rs571312	A	C	0.052	0.007	A	C	1.84×10^{-3}	5.40×10^{-3}	7.33×10^{-1}	A	C	6.61×10^{-3}	1.14×10^{-2}	5.61×10^{-1}	A	C	2.18×10^{-2}	1.66×10^{-2}	1.90×10^{-1}
rs61765651	C	T	0.047	0.008	T	C	-9.01×10^{-3}	6.10×10^{-3}	1.40×10^{-1}	T	C	-1.92×10^{-2}	1.26×10^{-2}	1.29×10^{-1}	T	C	-1.16×10^{-2}	1.87×10^{-2}	5.33×10^{-1}
rs62107261	T	C	0.121	0.018	C	T	5.41×10^{-3}	1.13×10^{-2}	6.33×10^{-1}	C	T	-3.36×10^{-2}	2.24×10^{-2}	1.34×10^{-1}	C	T	-2.13×10^{-2}	3.30×10^{-2}	5.19×10^{-1}
rs62500888	A	G	0.037	0.006	G	A	4.09×10^{-3}	4.59×10^{-3}	3.72×10^{-1}	G	A	8.95×10^{-3}	9.48×10^{-3}	3.45×10^{-1}	G	A	1.70×10^{-2}	1.39×10^{-2}	2.19×10^{-1}
rs7138803	A	G	0.072	0.006	A	G	4.76×10^{-4}	4.71×10^{-3}	9.19×10^{-1}	A	G	2.58×10^{-2}	9.77×10^{-3}	8.37×10^{-3}	A	G	2.66×10^{-2}	1.43×10^{-2}	6.31×10^{-2}
rs76227980	C	T	0.14	0.023	T	C	8.37×10^{-3}	1.69×10^{-2}	6.20×10^{-1}	T	C	-4.07×10^{-2}	3.82×10^{-2}	2.87×10^{-1}	T	C	-5.94×10^{-2}	5.64×10^{-2}	2.93×10^{-1}
rs7719067	A	G	0.036	0.006	G	A	-3.33×10^{-3}	4.62×10^{-3}	4.72×10^{-1}	G	A	4.18×10^{-3}	9.63×10^{-3}	6.64×10^{-1}	G	A	-4.36×10^{-4}	1.42×10^{-2}	9.75×10^{-1}
rs939584	T	C	0.092	0.008	T	C	1.02×10^{-2}	6.04×10^{-3}	9.00×10^{-2}	T	C	1.62×10^{-2}	1.25×10^{-2}	1.95×10^{-1}	T	C	4.38×10^{-2}	1.85×10^{-2}	1.78×10^{-2}

Table S2: The IVs information in the replication MR analysis

SNP	Childhood BMI				COVID–19 susceptibility					COVID–19 hospitalization					COVID–19 severity				
	EA	NEA	Beta	SE	EA	NEA	Beta	Se	P	EA	NEA	Beta	Se	P	EA	NEA	Beta	Se	P
rs11676272	G	A	0.068	0.007	G	A	5.30×10^{-3}	4.54×10^{-3}	2.42×10^{-1}	G	A	2.21×10^{-2}	9.41×10^{-3}	1.89×10^{-2}	G	A	3.37×10^{-4}	1.38×10^{-2}	9.81×10^{-1}
rs12041852	G	A	0.046	0.007	A	G	-4.25×10^{-4}	4.65×10^{-3}	9.27×10^{-1}	A	G	-1.49×10^{-2}	1.02×10^{-2}	1.41×10^{-1}	A	G	4.69×10^{-3}	1.48×10^{-2}	7.52×10^{-1}
rs12429545	A	G	0.076	0.01	A	G	8.05×10^{-4}	6.93×10^{-3}	9.08×10^{-1}	A	G	1.21×10^{-2}	1.53×10^{-2}	4.28×10^{-1}	A	G	2.95×10^{-2}	2.25×10^{-2}	1.89×10^{-1}
rs13130484	T	C	0.067	0.007	T	C	1.06×10^{-2}	4.73×10^{-3}	2.55×10^{-2}	T	C	2.82×10^{-2}	1.01×10^{-2}	5.16×10^{-3}	T	C	2.57×10^{-2}	1.46×10^{-2}	7.83×10^{-2}
rs13253111	A	G	0.042	0.007	G	A	4.10×10^{-3}	4.59×10^{-3}	3.72×10^{-1}	G	A	8.98×10^{-3}	9.48×10^{-3}	3.44×10^{-1}	G	A	1.68×10^{-2}	1.39×10^{-2}	2.25×10^{-1}
rs13387838	A	G	0.139	0.025	A	G	-1.67×10^{-2}	1.75×10^{-2}	3.41×10^{-1}	A	G	-6.74×10^{-2}	3.46×10^{-2}	5.17×10^{-2}	A	G	-7.31×10^{-3}	4.95×10^{-2}	8.83×10^{-1}
rs1421085	C	T	0.059	0.007	C	T	1.48×10^{-3}	4.61×10^{-3}	7.48×10^{-1}	C	T	1.68×10^{-2}	9.54×10^{-3}	7.78×10^{-2}	C	T	3.88×10^{-2}	1.39×10^{-2}	5.37×10^{-3}
rs3829849	T	C	0.041	0.007	T	C	9.81×10^{-3}	4.74×10^{-3}	3.85×10^{-2}	T	C	-2.46×10^{-3}	9.91×10^{-3}	8.04×10^{-1}	T	C	-4.52×10^{-3}	1.46×10^{-2}	7.57×10^{-1}
rs4854349	C	T	0.09	0.009	C	T	1.05×10^{-2}	6.14×10^{-3}	8.78×10^{-2}	C	T	1.92×10^{-2}	1.33×10^{-2}	1.48×10^{-1}	C	T	3.62×10^{-2}	1.94×10^{-2}	6.25×10^{-2}
rs543874	G	A	0.077	0.009	G	A	3.75×10^{-3}	5.79×10^{-3}	5.17×10^{-1}	G	A	1.03×10^{-2}	1.23×10^{-2}	4.02×10^{-1}	G	A	2.94×10^{-2}	1.82×10^{-2}	1.07×10^{-1}
rs6567160	C	T	0.05	0.008	C	T	1.76×10^{-3}	5.44×10^{-3}	7.47×10^{-1}	C	T	6.07×10^{-3}	1.15×10^{-2}	5.97×10^{-1}	C	T	2.16×10^{-2}	1.67×10^{-2}	1.96×10^{-1}
rs7132908	A	G	0.066	0.008	A	G	1.98×10^{-3}	4.65×10^{-3}	6.70×10^{-1}	A	G	2.50×10^{-2}	9.66×10^{-3}	9.72×10^{-3}	A	G	2.59×10^{-2}	1.42×10^{-2}	6.77×10^{-2}
rs7550711	T	C	0.105	0.019	T	C	3.90×10^{-3}	1.28×10^{-2}	7.61×10^{-1}	T	C	3.25×10^{-2}	2.93×10^{-2}	2.68×10^{-1}	T	C	4.45×10^{-2}	4.49×10^{-2}	3.22×10^{-1}
rs8092503	G	A	0.045	0.008	G	A	3.06×10^{-3}	5.40×10^{-3}	5.71×10^{-1}	G	A	1.38×10^{-2}	1.17×10^{-2}	2.39×10^{-1}	G	A	2.81×10^{-2}	1.74×10^{-2}	1.07×10^{-1}
rs987237	G	A	0.062	0.009	G	A	6.11×10^{-3}	5.79×10^{-3}	2.91×10^{-1}	G	A	2.87×10^{-2}	1.23×10^{-2}	1.96×10^{-2}	G	A	2.72×10^{-2}	1.82×10^{-2}	1.35×10^{-1}

Table S3: The casual association between childhood BMI and COVID-19 in the replication MR analysis

Outcomes	Methods	Number of SNPs	OR	95% CI	*P*-value	P for intercept of MR-Egger	P for Cochran's Q test	P for MR-PRESSO global test
COVID-19 susceptibility								
	IVW (fixed model)	15	1.06	1.02, 1.11	0.007		0.732	
	MR-Egger	15	1.05	0.88, 1.25	0.572	0.897		
	Weighted median	15	1.05	0.99, 1.12	0.109			
	Simple median	15	1.04	0.97, 1.10	0.251			
	Maximum-likelihood method	15	1.06	1.02, 1.11	0.007			
	MR-PRESSO (0 outliers)	15	1.06	1.02, 1.10	0.008			0.755
COVID-19 hospitalization								
	IVW (fixed model)	15	1.33	1.16, 1.53	<0.001		0.157	
	MR-Egger	15	1.80	1.05, 3.10	0.298	0.971		
	Weighted median	15	1.47	1.22, 1.78	<0.001			
	Simple median	15	1.47	1.22, 1.79	<0.001			
	Maximum-likelihood method	15	1.34	1.17, 1.55	<0.001			
	MR-PRESSO (0 outliers)	15	1.33	1.16, 1.53	0.001			0.196
COVID-19 severity								
	IVW (fixed model)	15	1.26	1.14, 1.38	<0.001		0.402	
	MR-Egger	15	1.26	0.81, 1.97	0.033	0.256		
	Weighted median	15	1.35	1.19, 1.53	<0.001			
	Simple median	15	1.33	1.16, 1.52	<0.001			
	Maximum-likelihood method	15	1.26	1.13, 1.41	<0.001			
	MR-PRESSO (0 outliers)	15	1.26	1.12, 1.40	0.001			0.430
